# Immune Dysfunction in Rett Syndrome Patients Revealed by High Levels of Serum Anti-N(Glc) IgM Antibody Fraction

**DOI:** 10.1155/2014/260973

**Published:** 2014-10-15

**Authors:** Anna Maria Papini, Francesca Nuti, Feliciana Real-Fernandez, Giada Rossi, Caterina Tiberi, Giuseppina Sabatino, Shashank Pandey, Silvia Leoncini, Cinzia Signorini, Alessandra Pecorelli, Roberto Guerranti, Solange Lavielle, Lucia Ciccoli, Paolo Rovero, Claudio De Felice, Joussef Hayek

**Affiliations:** ^1^Laboratory of Peptide and Protein Chemistry and Biology, University of Florence, Via della Lastruccia 13, 50019 Sesto Fiorentino, Italy; ^2^Department of Chemistry “Ugo Schiff”, University of Florence, 50019 Sesto Fiorentino, Italy; ^3^Laboratory of PeptLab-SOSCO-EA4505, University of Cergy-Pontoise, 5 mail Gay Lussac, 95031 Cergy-Pontoise CEDEX, France; ^4^Toscana Biomarkers Srl, 53100 Siena, Italy; ^5^Section of Pharmaceutical and Nutraceutical Sciences, Department NeuroFarBa, University of Florence, 50019 Sesto Fiorentino, Italy; ^6^Child Neuropsychiatry Unit, University Hospital, Azienda Ospedaliera Universitaria Senese (AOUS), 53100 Siena, Italy; ^7^Department of Molecular and Developmental Medicine, University of Siena, 53100 Siena, Italy; ^8^Department of Medical Biotechnologies, University of Siena, 53100 Siena, Italy; ^9^Clinical Pathology Laboratory Unit, University Hospital, AOUS, 53100 Siena, Italy; ^10^Laboratory of BioMolecules, Sorbonne Université UPMC Paris 06, CNRS-ENS, 75005 Paris, France; ^11^Neonatal Intensive Care Unit, University Hospital, AOUS, 53100 Siena, Italy

## Abstract

Rett syndrome (RTT), a neurodevelopmental disorder affecting exclusively (99%) female infants, is associated with loss-of-function mutations in the gene encoding methyl-CpG binding protein 2 (*MECP2*) and, more rarely, cyclin-dependent kinase-like 5 (*CDKL5*) and forkhead box protein G1 (*FOXG1*). In this study, we aimed to evaluate the function of the immune system by measuring serum immunoglobulins (IgG and IgM) in RTT patients (*n* = 53) and, by comparison, in age-matched children affected by non-RTT pervasive developmental disorders (non-RTT PDD) (*n* = 82) and healthy age-matched controls (*n* = 29). To determine immunoglobulins we used both a conventional agglutination assay and a novel ELISA based on antibody recognition by a surrogate antigen probe, CSF114(Glc), a synthetic *N*-glucosylated peptide. Both assays provided evidence for an increase in IgM titer, but not in IgG, in RTT patients relative to both healthy controls and non-RTT PDD patients. The significant difference in IgM titers between RTT patients and healthy subjects in the CSF114(Glc) assay (*P* = 0.001) suggests that this procedure specifically detects a fraction of IgM antibodies likely to be relevant for the RTT disease. These findings offer a new insight into the mechanism underlying the Rett disease as they unveil the possible involvement of the immune system in this pathology.

## 1. Introduction

Rett syndrome (RTT), an X-linked neurodevelopment disorder affecting almost exclusively females, is associated with a single monogenic mutation (methyl-CpG binding protein 2,* MeCP2*) in up to 95% of cases [1], more rarely by mutations in cyclin-dependent kinase-like 5 (*CDKL5*) [2], and forkhead box protein G1 (*FOXG1*) gene [3]. RTT syndrome, formerly placed in the heterogeneous group of autism spectrum disorders (ASD), is now regarded as a distinct pathological entity. The recently issued clinical criteria for RTT diagnosis have clearly defined the core features of the disease. Approximately 80% of RTT patients show the typical clinical features: early neurological regression, followed by loss of acquired cognitive, social, and motor skills in a typical four-stage neurological regression, together with development of autistic behavior [4].

RTT patients with atypical clinical presentation usually harbor* CDKL5* (early seizure variant, ESV) or* FOXG1* (congenital variant, CV) mutations, while the preserved speech variant (PSV) is usually linked to* MeCP2* mutations [5]. Despite almost two decades of research into the functions and role of MeCP2, surprisingly little is known about the mechanisms leading from MeCP2 deficiency to disease expression.

Research, focused largely on* in vivo* functions of MeCP2 in transgenic mice lacking the* Mecp2 *gene, has provided evidence for a repressive action of the transcription factor on neuronal genes associated with dendrite arborization as well as other neuronal functions [6].

In contrast, evidences indicate that, in central nervous systems (CNS) cells other than neurons, as astrocytes and microglia,* MeCP2 *ablation impairs upregulation of genes, which support dendritic arborization, and the inherent phagocytic activity of microglia [7, 8]. In light of demonstrated involvement of microglia in disease progression in* Mecp2*-null mice, we hypothesize the coexistence of a perturbation of the immune system in RTT patients. In particular, a derangement of microglia immune responsiveness might be likely to occur in these patients, as neuroinflammation is a powerful modulator of the CNS immune system.

In pathological CNS conditions, such as RTT and others (e.g., multiple sclerosis, MS) in which neither the antibodies nor the antigens involved in the disease are known, the detection of putative antibodies in biological fluids may be achieved by employing a library of unnatural peptides, which, by virtue of their conformation and posttranslational modifications (e.g.,* N*-glucosylation), recognize and bind to the relevant antibodies [9, 10]. We have previously used this approach to characterize antibodies present in multiple sclerosis patients' serum by applying a further refinement, that is, the* N*-glucosylation, to the molecule of the surrogate peptide antigen [11, 12]. The rationale for this chemical modification stems from the recognition that* N*-glucosylation is a pervasive feature of biomolecules occurring in CNS pathologies characterized by neuronal disruption. In this work, we took advantage of prior experience gained on MS antibodies using the CSF114(Glc) peptide antigenic probe, with the aim of investigating the presence of antibodies in serum of RTT patients, and by comparison to healthy controls and non-RTT pervasive developmental disorders patients.

## 2. Material and Methods

### 2.1. Patients

In this study, a total of 164 patients were enrolled. This population consisted of three clearly distinguishable groups: the RTT syndrome group versus non-RTT pervasive developmental disorders (non-RTT PDD) group on the basis of the clinical features and the presence of mutated RTT-related genes and healthy, age-matched controls. The RTT group consisted of 53 patients (mean age 12.8 ± 10.7 years) who were referred to our clinical unit (a referral center for a vast region in central Italy) subdivided into *n* = 41 (77.4%) with classical clinical presentation with proven* MeCP2* gene mutation and *n* = 12 (12.6%) atypical presentation (of whom *n* = 9, *n* = 2, and *n* = 1 linked to* CDKL5*,* FOXG1*, and* MeCP2* mutations, resp.). These patients were hospitalized for 1 week every 6 months, in the Child Neuropsychiatric Unit, University Hospital Azienda Ospedaliera Universitaria Senese of Siena (Italy), during the course of the study. Criteria for inclusion in the study were clinical diagnosis of RTT syndrome coupled with positive identification for the presence/absence of mutated* MeCP2*,* CDKL5*, or* FOXG1* genes. The age-matched non-RTT PDD group consisted of 82 patients (mean age 13.0 ± 9.5 years), as diagnosed on the basis of well-established criteria. They were recruited from those attending our unit for routine clinical follow-up. Blood samplings in the patients' group were performed during the routine follow-up study at hospital admission, while the samples from the control group were carried out during routine health checks, sports, or blood donations, obtained during the periodic clinical checks. The healthy control subjects were age-matched.

Recruitment of patients was performed in a diagnostic workup context. Patients were selected randomly and not previously tested for immune reactivity by ELISA. This study was approved by the Institutional Review Board of Azienda Ospedaliera Universitaria Senese. Parents, tutors, or guardians of all the participants provided their written informed consent for the minors to participate in this study. The study design, methods, and consent procedure were approved by the Institutional Review Board of Azienda Ospedaliera Universitaria Senese. All the data used in this study was anonymized.

### 2.2. Total Plasma Immunoglobulins

Determination of total plasma IgG and IgM was performed using the Cobas 6000 system (Roche Diagnostics). Briefly, the Roche test is based on the principle of agglutination immunoassay where anti-Ig antibodies react with the antigen in the sample, forming an antigen-antibody complex, which, after agglutination, is measured turbidimetrically. It is important to note that this method is standard in the clinical chemistry laboratories and is independent from the recognition of the synthetic* N*-glucosylated peptide epitope.

### 2.3. Determination of Serum Antibodies by SP-ELISA

Antibody responses were determined in SP-ELISA. Ninety-six well activated polystyrene ELISA plates (NUNC Maxisorp Sigma) were coated with 1 *μ*g per 100 *μ*L of glycopeptide in carbonate buffer 0.05 M (pH 9.6) and incubated at +4°C overnight. After five washes with saline containing 0.05% Tween 20, nonspecific binding sites were blocked by fetal bovine serum (FBS), 10% in saline Tween 20 (100 *μ*L per well) at room temperature for 60 min. Sera diluted from 1 : 100 to 1 : 10,000 were applied at +4°C overnight in saline/Tween 20/10% FBS. After five washes, we added to each well 100 *μ*L of alkaline phosphatase conjugated antihuman IgM (diluted 1 : 200 in saline/Tween 20/FBS; Sigma) or IgG (diluted 1 : 8000 in saline/Tween 20/FBS; Sigma). After 3 h at room temperature incubation and five washes, 100 *μ*L of substrate solution consisting of 1 mg mL^−1^ p-nitrophenyl phosphate (Sigma) in 10% diethanolamine buffer was applied. After 30 min, the reaction was stopped with 1 M NaOH (50 *μ*L), and the absorbance was read in a multichannel ELISA reader (Tecan Sunrise) at 405 nm. ELISA plates, coating conditions, reagent dilutions, buffers, and incubation times were tested in preliminary experiments. Antibody levels are expressed as absorbance in arbitrary units at 405 nm (sample dilution 1 : 100).

### 2.4. Data Analysis

All variables were tested for normal distribution (D'Agostino-Pearson test) and data were presented as either means ± standard deviation (SD) for normally distributed variables or medians and interquartile range values for non-normally distributed data. Differences between groups were evaluated. The efficiency of anti-CSF114(Glc) IgM in discriminating RTT from either non-RTT PDD patients or healthy control subjects was evaluated using a receiver operating characteristic (ROC) curve analysis. Two-tailed* P* values of less than 0.05 were considered significant. Correction for multiple comparisons was made (Bonferroni's correction). The MedCalc version 12.1.4 statistical software package (MedCalc Software, Mariakerke, Belgium) was used.

## 3. Results

**Figure 1:**
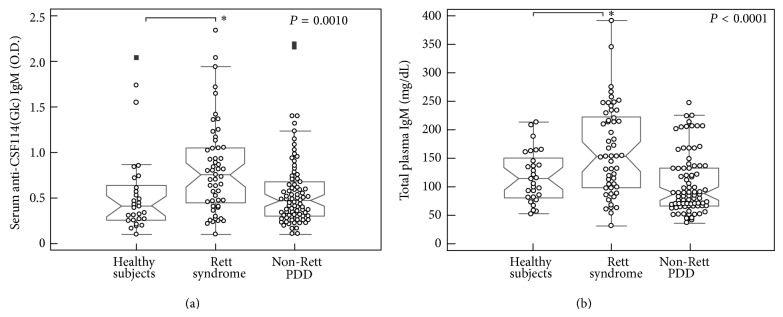
Elevated autoantibodies anti-CSF114(Glc) and total IgM titers in Rett syndrome. (a) Anti-CSF114(Glc) serum IgM antibody titers by the* N*-glucopeptide-based immunoenzymatic assay: comparison between RTT and non-RTT PDD patients. (b) Total plasma IgM levels detected by the conventional agglutination assay (Roche Cobas 6000). Data are plotted as notched box dot plots with medians and interquartile range values. *P* values refer to nonparametric Kruskal-Wallis analysis of variance. Solid rectangles indicate outlier values, ^*^ *P* < 0.05.

The presence of putative serum antibodies (Abs) in the selected patients' population and healthy subjects was evaluated by both a conventional agglutination procedure and solid-phase ELISA, using an* N*-glucosylated *β*-turn peptide probe, termed CSF114(Glc). The IgM antibody titers clearly segregate the two patient groups (Figure 1).

**Figure 2:**
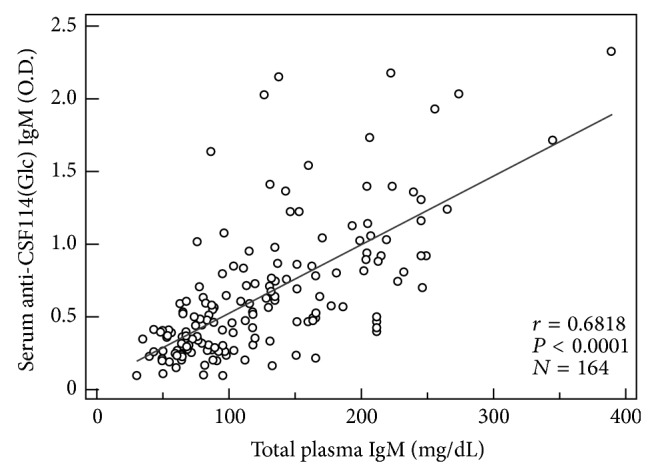
Correlation between anti-CSF114(Glc) serum IgM antibody titers and total plasma IgM levels in RTT patients' sera.

In fact, the individual IgM antibody titers found in RTT patients were significantly higher than those in the control groups, that is, both healthy subjects and non-RTT PDD patients. These changes of IgM antibodies were detected either by assaying antibodies through an agglutination assay routinely used in the hospital laboratory (Roche Cobas 6000) or by the* N*-glucopeptide-based immunoenzymatic assay based on the novel CSF114(Glc) antigen probe (Figures 1(a) and 1(b), *P* < 0.05). Although the two assays are not directly comparable, since they provide different absolute values of antibody titers, nonetheless they both indicate a consistent relative increase of serum IgM in the RTT group. Moreover, we observed a linear correlation between anti-CSF114(Glc) serum IgM antibody titers and total plasma IgM levels in RTT patients (Figure 2).

Whereas the agglutination assay detects the total serum antibody population, the CSF114(Glc) identifies a small fraction of this population. These observed differences are, in our view, evidence that CSF114(Glc) captures specific antibodies associated with the disease.

**Figure 3:**
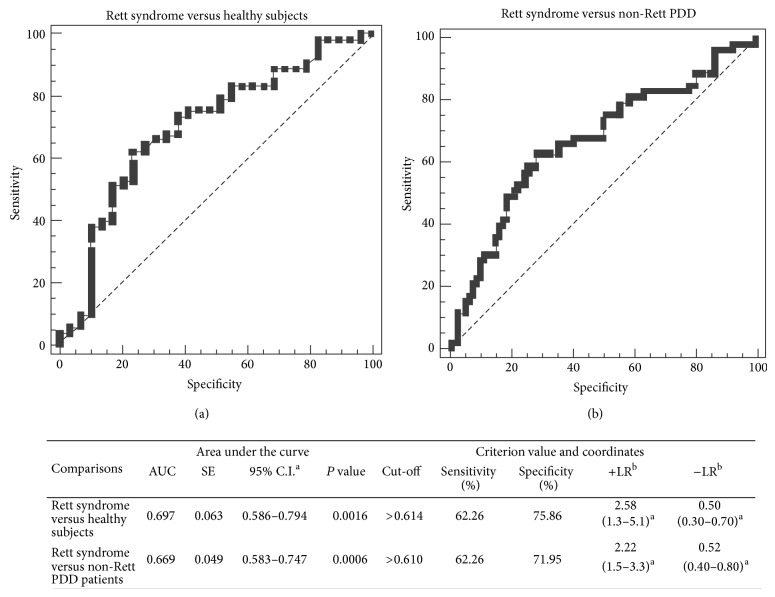
Receiver operating characteristic (ROC) analysis for anti-CSF114(Glc) serum IgM antibody titer. ROC curve discriminates RTT patients versus healthy subjects (a) and RTT patients from non-RTT PDD patients (b). Abbreviations list: AUC: area under the curve; SE: standard error; C.I: confidence intervals; L.R.: likelihood ratio. ^a^95% confidence interval; ^b^likelihood ratio.

Receiver operating characteristic (ROC) curves indicated that anti-CSF114(Glc) titers are able to discriminate RTT syndrome from either non-RTT PDD patients or control subjects (Figure 3).

At variance, while the CSF114(Glc) antigen failed to detect significant differences in auto IgG auto-antibody titers among the three groups (RTT vs. non-RTT PDD vs. healthy controls), the total plasma IgG titer in the RTT population was significantly decreased. (Supplementary Figure 1 in Supplementary Material available online at http://dx.doi.org/10.1155/2014/260973).

## 4. Discussion

This report describes the changes observed in serum immunoglobulins IgM and IgG, in RTT patients in comparison to healthy subjects and patients afflicted by diffused autistic traits (i.e., non-RTT PDD). The two patients groups were defined on the basis of the presence of known gene mutations (mainly* MeCP2*) for the RTT group and of well-established diagnostic criteria for the non-RTT PDD group. Although a number of studies have addressed the relationship between RTT and immune system dysfunction, these have mainly focused on their influence on peripheral immune cells [8], although two early reports investigated humoral immunity in RTT patients, observing evidences of immune system activation [13, 14]. Here, we demonstrate that RTT patients show a consistent and highly significant increase of the serum IgM fraction relative to both healthy controls and non-RTT PDD patients group. This was shown by determining the serum IgM titer by two independent assay procedures. We assayed immunoglobulin by a conventional, widely used, hospital procedure based on agglutination and as a complementary assay, an antibody recognition procedure based on a surrogate antigen, termed CSF114(Glc), a peptide bearing* N*-glucosylation on its moiety. In our view, the IgM fraction recognized by CSF114(Glc), tiny in comparison to the large one detected by the agglutination procedure, represents an antibody pool of pathophysiological relevance for this disease. Supporting this view is the remarkable specificity of interaction between CSF114(Glc) and IgM antibodies demonstrated by the receiver operating curve, widely considered a test of accuracy for discriminating between diseased patients and normal subjects. Thus, it appears that the RTT patients group can be distinguished with confidence on the basis of serum IgM titer detected by the CSF114(Glc)-based assay. It is of interest that the large serum IgM increases, noted here, were also observed by us and others, in a subset of multiple sclerosis patients as well as in meningitis and encephalitis patients ([14] and references therein). It is plausible that the IgM changes, noted in these CNS pathologies, reflect a common underlying mechanism, most probably a pervasive neuroinflammation. We suggest that the association between neuroinflammation and the observed serum IgM rise might be causal to the RTT disease initiation and/or progression. The question arises whether the neuronal derangement elicits the immune response, or conversely, the immune dysfunction might trigger the neuroinflammation. Although the present observations do not answer this “chicken or egg question,” there are indications that IgM overexpression represents primarily a line of defense against noxious stimuli.

In contrast to IgM fraction, we found small changes in the serum IgG of RTT patients when assayed with the agglutination assay, while the CSF114(Glc) assay performed poorly in detecting IgG antibodies.

Further considerations concern the growing research area of epigenetics, which reportedly appears to be involved in autoimmune diseases. These are generally considered to be caused by a combination of epigenetic modification, deregulated immunomodulation, and environmental factors. Several single-nucleotide polymorphisms (SNPs) at an X chromosome locus harboring the* MeCP2* gene have previously been shown to be associated with susceptibility to systemic lupus erythematosus, as well as with a variety of systemic autoimmune diseases [15]. Recent reports on experimental models in mice have placed microglia at the center stage of the RTT progression, as the Mecp2*-*deficient mice reproduce most of the human RTT phenotype features [7, 16]. Because microglia exert a prominent immune surveillance role in the CNS, it is likely that the large serum IgM increase reflects and signals a severe disturbance of immune regulation in brain [17].

In conclusion, the results of this work contribute to elucidate that the two disease conditions such as Rett syndrome and the diffuse autism, seemingly contiguous as they share some behavioral traits, are in fact dramatically different for their severity, life-span expectancy, and, as we now learn, immune system derangement.

## Supplementary Material

Supplementary Figure 1: Decreased total IgG along with comparable IgG autoantibodies anti-CSF114 (Glc) titers in Rett syndrome. (a) Anti-CSF114(Glc) serum IgG antibody titers by the N-glucopeptide-based immunoenzymatic assay. (b) Total plasma IgG levels detected by the conventional agglutination assay (Roche Cobas 6000). Data are plotted as notched box dot plots with medians and inter-quartile range values. P values refer to non-parametric Kruskal-Wallis analysis of variance. Solid rectangles indicate outlier values. ∗*P*<0.05.

## References

[1] Chahrour M., Zoghbi H. Y. (2007). The story of Rett syndrome: from clinic to neurobiology. *Neuron*.

[2] Mari F., Azimonti S., Bertani I., Bolognese F., Colombo E., Caselli R., Scala E., Longo I., Grosso S., Pescucci C., Ariani F., Hayek G., Balestri P., Bergo A., Badaracco G., Zappella M., Broccoli V., Renieri A., Kilstrup-Nielsen C., Landsberger N. (2005). CDKL5 belongs to the same molecular pathway of MeCP2 and it is responsible for the early-onset seizure variant of Rett syndrome. *Human Molecular Genetics*.

[3] Ariani F., Hayek G., Rondinella D., Artuso R., Mencarelli M. A., Spanhol-Rosseto A., Pollazzon M., Buoni S., Spiga O., Ricciardi S., Meloni I., Longo I., Mari F., Broccoli V., Zappella M., Renieri A. (2008). FOXG1 is responsible for the congenital variant of Rett syndrome. *The American Journal of Human Genetics*.

[4] Hagberg B. (2002). Clinical manifestations and stages of Rett syndrome. *Mental Retardation and Developmental Disabilities Research Reviews*.

[5] Neul J. L., Kaufmann W. E., Glaze D. G., Christodoulou J., Clarke A. J., Bahi-Buisson N., Leonard H., Bailey M. E. S., Schanen N. C., Zappella M., Renieri A., Huppke P., Percy A. K. (2010). Rett syndrome: revised diagnostic criteria and nomenclature. *Annals of Neurology*.

[6] Chahrour M., Sung Y. J., Shaw C., Zhou X., Wong S. T. C., Qin J., Zoghbi H. Y. (2008). MeCP2, a key contributor to neurological disease, activates and represses transcription. *Science*.

[7] Derecki N. C., Cronk J. C., Lu Z., Xu E., Abbott S. B. G., Guyenet P. G., Kipnis J. (2012). Wild-type microglia arrest pathology in a mouse model of Rett syndrome. *Nature*.

[8] Derecki N. C., Privman E., Kipnis J. (2010). Rett syndrome and other autism spectrum disorders—brain diseases of immune malfunction. *Molecular Psychiatry*.

[9] Lolli F., Mulinacci B., Carotenuto A., Bonetti B., Sabatino G., Mazzanti B., D'Ursi A. M., Novellino E., Pazzagli M., Lovato L., Alcaro M. C., Peroni E., Pozo-Carrero M. C., Nuti F., Battistini L., Borsellino G., Chelli M., Rovero P., Papini A. M. (2005). An N-glucosylated peptide detecting disease-specific autoantibodies, biomarkers of multiple sclerosis. *Proceedings of the National Academy of Sciences of the United States of America*.

[10] Reddy M. M., Wilson R., Wilson J., Connell S., Gocke A., Hynan L., German D., Kodadek T. (2011). Identification of candidate IgG biomarkers for alzheimer's disease via combinatorial library screening. *Cell*.

[11] Carotenuto A., D'Ursi A. M., Mulinacci B., Paolini I., Lolli F., Papini A. M., Novellino E., Rovero P. (2006). Conformation-activity relationship of designed glycopeptides as synthetic probes for the detection of autoantibodies, biomarkers of multiple sclerosis. *Journal of Medicinal Chemistry*.

[12] Pandey S., Dioni I., Lambardi D., Real-Fernandez F., Peroni E., Pacini G., Lolli F., Seraglia R., Papini A. M., Rovero P. (2013). Alpha actinin is specifically recognized by multiple sclerosis autoantibodies isolated using an N-glucosylated peptide epitope. *Molecular and Cellular Proteomics*.

[13] Fiumara A., Sciotto A., Barone R., D'Asero G., Munda S., Parano E., Pavone L. (1999). Peripheral lymphocyte subsets and other immune aspects in Rett syndrome. *Pediatric Neurology*.

[14] Reichelt K. L., Skjeldal O. (2006). IgA antibodies in Rett syndrome. *Autism*.

[15] Han T.-U., Cho S.-K., Kim T., Joo Y. B., Bae S.-C., Kang C. (2013). Association of an activity-enhancing variant of IRAK1 and an MECP2-IRAK1 haplotype with increased susceptibility to rheumatoid arthritis. *Arthritis & Rheumatism*.

[16] Lioy D. T., Garg S. K., Monaghan C. E., Raber J., Foust K. D., Kaspar B. K., Hirrlinger P. G., Kirchhoff F., Bissonnette J. M., Ballas N., Mandel G. (2011). A role for glia in the progression of Rett-syndrome. *Nature*.

[17] Aguzzi A., Barres B. A., Bennett M. L. (2013). Microglia: scapegoat, saboteur, or something else?. *Science*.

