# Supplementary material for: Immune Dysfunction in Rett Syndrome Patients Revealed by High Levels of Serum Anti-N(Glc) IgM Antibody Fraction
Source: J Immunol Res. 2014 Oct 15;2014:260973. doi: 10.1155/2014/260973 (PMC4214166; doi:10.1155/2014/260973)
Supplement: Supplementary file 1 — Supplementary Figure 1: Decreased total IgG along with comparable IgG autoantibodies anti-CSF114 (Glc) titers in Rett syndrome. (a) Anti-CSF114(Glc) serum IgG antibody titers by the N-glucopeptide-based immunoenzymatic assay. (b) Total plasma IgG levels detected by the conventional agglutination assay (Roche Cobas 6000). Data are plotted as notched box dot plots with medians and inter-quartile range values. P values refer to non-parametric Kruskal-Wallis analysis of variance. Solid rectangles indicate outlier values. ∗P<0.05. [file 260973.f1.pdf]

Supporting Information

Immune dysfunction in Rett syndrome patients revealed by high levels of serum anti-N(Glc) IgM antibody fraction

Papini et al.

The CSF114(Glc) antigen failed to detect a significant IgG serum antibody population in both patient groups and healthy controls, the majority of the IgG titers being close to the detection limit (Supplementary Figure 1).

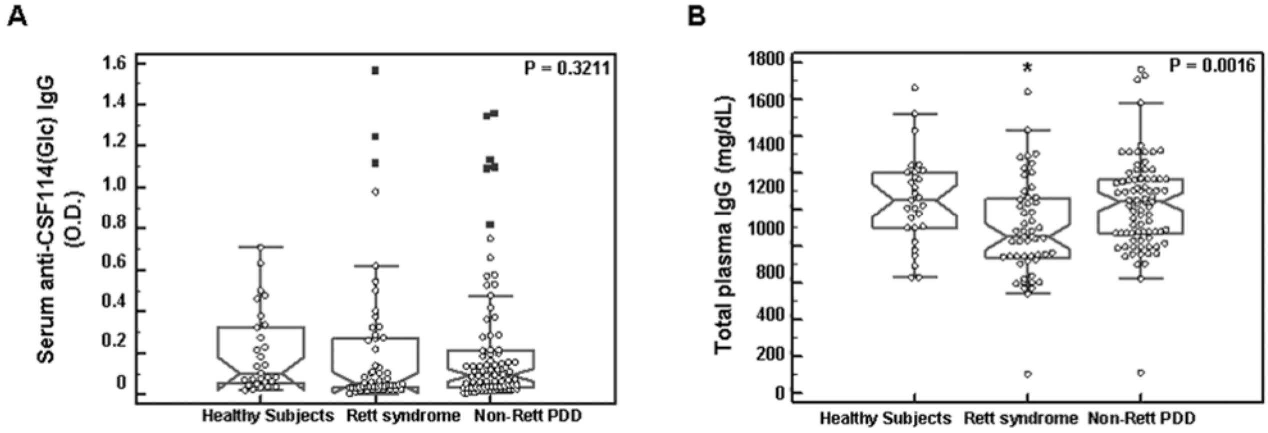

Supplementary Figure 1. Decreased total IgG along with comparable IgG autoantibodies anti-CSF114(Glc) titers in Rett syndrome. (a) Anti-CSF114(Glc) serum IgG antibody titers by the N-glucopeptide-based immunoenzymatic assay. (b) Total plasma IgG levels detected by the conventional agglutination assay (Roche Cobas 6000). Data are plotted as notched box dot plots with medians and inter-quartile range values. P values refer to non-parametric Kruskal-Wallis analysis of variance. Solid rectangles indicate outlier values. * $P < 0.05$.
